# Supplementary material for: Competitive plasticity to reduce the energetic costs of learning
Source: PLoS Comput Biol. 2024 Oct 28;20(10):e1012553. doi: 10.1371/journal.pcbi.1012553 (PMC11542811; doi:10.1371/journal.pcbi.1012553)
Supplement: S1 Text — Single file with 2 supplementary figures: Supplementary figure 1: Influence of various assumptions on energy saving. Supplementary figure 2: Relation between train and test error comparing plain backprop to competitive updating. (PDF) [file pcbi.1012553.s001.pdf]

Supplementary Information:
Competitive plasticity to reduce the energetic costs of learning

Mark C.W. van Rossum and Aaron Pache

October 22, 2024

University of Nottingham, Nottingham, UK

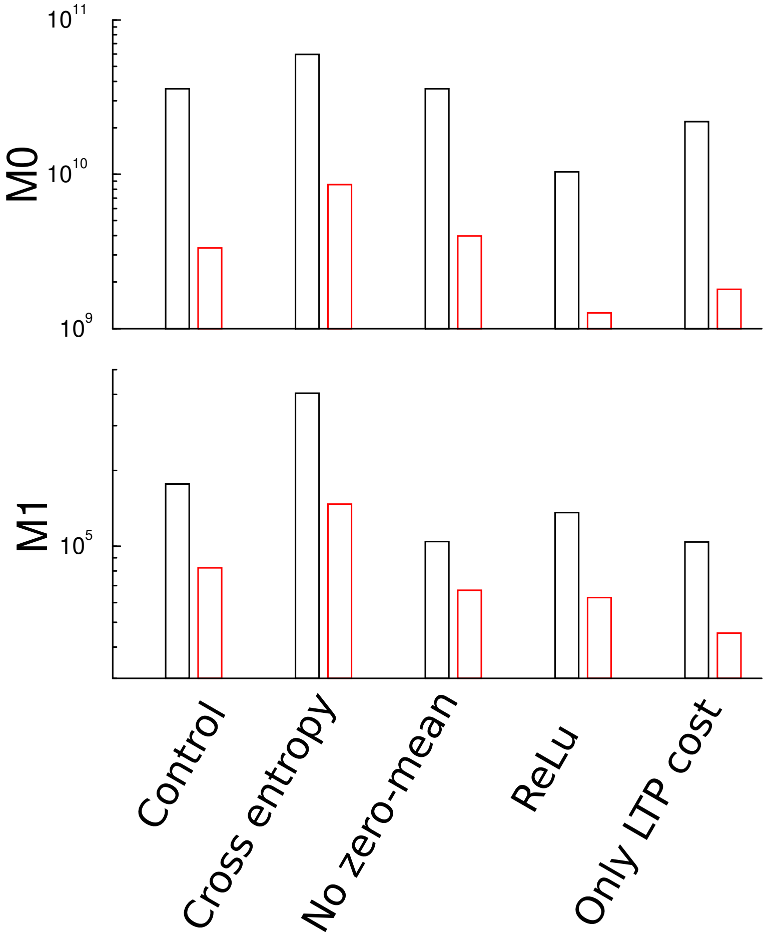

Supplementary Figure A: Influence of various assumptions on energy saving. While the energy levels change, the amount of savings achievable remains similar. Each pair of bars shows the energy required for a network with 2500 hidden units with unconstrained plasticity (black), and when using the optimal fraction of plastic synapses with a fixed mask (red). From left to right, **Control:** as in main text for comparison; **Cross entropy:** training on cross entropy loss function; **No zero-mean:** without zero-meaning the data; **ReLU:** using linear rectifying (ReLU) units in the hidden layer; **Only LTP cost:** only positive weight changes cost energy, negative changes are free.

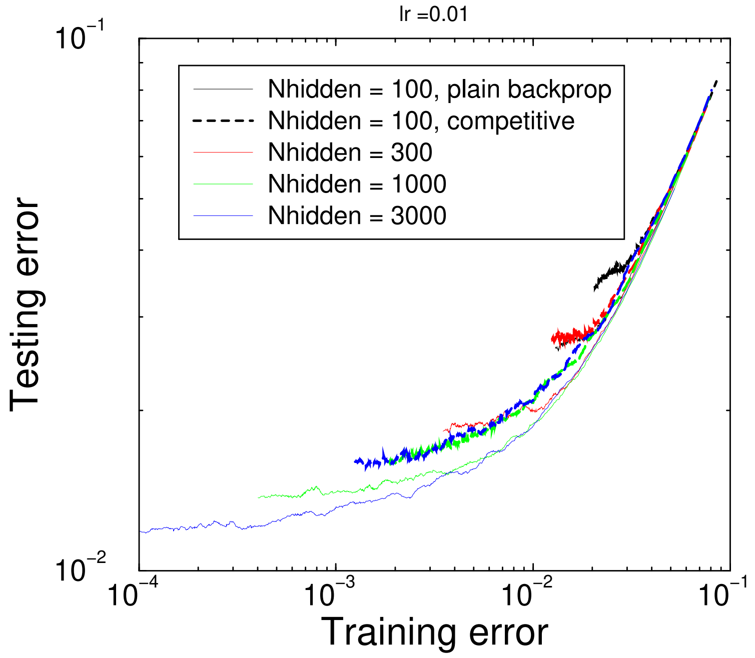

Supplementary Figure B: Relation between train and test error comparing plain backprop (thin curves) to competitive updating (thick dashed). Networks were trained for 30 epochs. In competitive updating only the 10×784 synapses with the largest update magnitude in the input-to-hidden layer were updated. In competitive updating, the testing error for a given training error is slightly higher. (learning rate 0.01)
