# Supplementary material for: Competitive plasticity to reduce the energetic costs of learning
Source: PLoS Comput Biol. 2024 Oct 28;20(10):e1012553. doi: 10.1371/journal.pcbi.1012553 (PMC11542811; doi:10.1371/journal.pcbi.1012553)
Supplement: Attachment — Submitted filename: reply.pdf [file pcbi.1012553.s002.pdf]

Dear Editor

We like to thank you and the reviewers for their positive comments. Please find our replies below. Note that my student Aaron Pache is now listed as co-author as he helped with the additional simulations presented in the supplement.

Reviewer #1:

I found the paper clear and a very enjoyable read. I am thus happy to recommend this paper for acceptance, leaving only a few minor questions and suggestions for the author below.

- Test accuracy and training error are usually strongly correlated on MNIST, but this is not always true. More as a sanity check, it would be good to perform some analysis of the training loss, and also to determine whether the energy saving methods converge to a minimizer (in particular for the high capacity, wide networks). Some methods achieve high test accuracy on MNIST, but cannot optimize the network well. In my experience, this is usually a sign of (big) problems, as these methods usually then falter on more difficult tasks.

We presume the reviewer meant high *training* accuracy, but poor test performance (otherwise it is not clear how to check this). In order to examine this we now include a new supplementary figure that show their relation.

- Do the results depend on how the network is initialized? There's a brief comment on input-to-hidden variance tuning for fixed hidden layer networks, but it might be worth looking at this in more detail. Note that network initialization can have a big impact on the learning dynamics (cf. "lazy training regime" and NTK theory).

This is indeed an interesting question. For the main results in the text, we extensively varied the initial weight variance and found a broad range over which energy was minimized. Too low and the network would be slow to start up, and too high and weights would have to shrink to learn the task. For the extreme learning machine (plasticity in output only), the choice is however critical. We are preparing a manuscript with a full analysis of that specific case, and now refer to that in the main text.

- In numerical optimization, the efficiency of an algorithm is typically analyzed in terms of its rate and order of convergence. It would be nice to discuss the intimate connection between the energy efficiency of learning as defined here and such concepts.

This sounds quite interesting, however we are unsure how this can be addressed in the current framework.

Reviewer #2:

I fully support the publication of this article, with only one minor revision: In the discussion, it is stated that the energy required for synaptic transmission is not considered, which could come across as confusing to a reader with a mostly biological background, as the M0 and M1 energy are specifically related to synaptic updates. I would suggest a "bridging" paragraph in the discussion or intro, which specifically spells out the way that energy use is being considered with respect to how energy is used at biological synapses. The update itself is what is costly - how akin is this to the ATP costs of filament assembly for a new spine, for

example?

We have now further worked this out. As stated, there is no consensus why learning is metabolically expensive, nor which aspect of synaptic plasticity is expensive. It is not even clear that the cost is synaptic. We explain this more extensively now.

Is there a cost associated with a decreased weight update?

Yes there is, but it does not matter; in the first supplementary figure, we show that this no qualitative effect on the possible savings.

We hope that the manuscript is now ready for publication.

Yours sincerely,

The authors
